# Avian influenza virus (H_5_N_1_); effects of physico-chemical factors on its survival

**DOI:** 10.1186/1743-422X-6-38

**Published:** 2009-03-28

**Authors:** Muhammad Akbar Shahid, Muhammad Abubakar, Sajid Hameed, Shamsul Hassan

**Affiliations:** 1 Poultry Research Institute, Shamsabad, Murree road, Punjab, Rawalpindi, Pakistan; 2 National Veterinary Laboratory, Park road, Islamabad, Pakistan; 3 University College of Veterinary and Animal Sciences, The Islamia University of Bahawalpur, Bahawalpur, Pakistan

## Abstract

Present study was performed to determine the effects of physical and chemical agents on infective potential of highly pathogenic avian influenza (HPAI) H5N1 (local strain) virus recently isolated in Pakistan during 2006 outbreak. H5N1 virus having titer 10^8.3 ^ELD_50_/ml was mixed with sterilized peptone water to get final dilution of 4HA units and then exposed to physical (temperature, pH and ultraviolet light) and chemical (formalin, phenol crystals, iodine crystals, CID 20, virkon^®^-S, zeptin 10%, KEPCIDE 300, KEPCIDE 400, lifebuoy, surf excel and caustic soda) agents. Harvested amnio-allantoic fluid (AAF) from embryonated chicken eggs inoculated with H5N1 treated virus (0.2 ml/egg) was subjected to haemagglutination (HA) and haemagglutination inhibition (HI) tests. H5N1 virus lost infectivity after 30 min at 56°C, after 1 day at 28°C but remained viable for more than 100 days at 4°C. Acidic pH (1, 3) and basic pH (11, 13) were virucidal after 6 h contact time; however virus retained infectivity at pH 5 (18 h), 7 and 9 (more than 24 h). UV light was proved ineffectual in inactivating virus completely even after 60 min. Soap (lifebuoy^®^), detergent (surf excel^®^) and alkali (caustic soda) destroyed infectivity after 5 min at 0.1, 0.2 and 0.3% dilution. All commercially available disinfectants inactivated virus at recommended concentrations. Results of present study would be helpful in implementing bio-security measures at farms/hatcheries levels in the wake of avian influenza virus (AIV) outbreak.

## Introduction

Poultry industry in Pakistan is facing various managemental problems along with infectious diseases including avian influenza (AI). This disease of highly pathogenic type was first reported in Pakistan in 1995, caused by subtype H7N3. Since then, various outbreaks of H7N3, H9N2 have been reported in various parts of the country which have inflicted heavy losses to the commercial poultry enterprises [[1,2] and [3]]. In February 2006, avian influenza virus (AIV) subtype H5N1 was for the first time found in two isolated commercial flocks in this country. Biosecurity measures, controlling poultry movements and inactivated vaccines were devised to combat the spread of newly introduced HPAIV H5N1 [4].

Avian influenza viruses by virtue of their infective potential pose a significant threat to human health. AIV subtypes, namely H5, H7 and H9, currently endemic in poultry in some regions of the world, have been shown capable of infecting humans [[5,6] and [7]]. Therefore, AI infections represent risk factors either for direct infection of humans from the avian host or for the consequences of genetic reassortment between a mammalian and an avian influenza virus, which could become the basis for a generation of a new pandemic virus for humans [8].

It is of crucial importance that AI infections in poultry are controlled to eradicate. International organizations have issued a list of recommendations aiming to control the AI in Asia [9]. The recommendations include implementation of risk reduction interventions such as restriction policies, stamping out, and under certain circumstances appropriate vaccination programmes. Secondary spread of AI is mainly caused through human-related activities such as the movement of staff, vehicles, equipment, and other fomites along with restocking of birds in establishments without following adequate biosecurity measures. It therefore implies that if disinfection of premises, footwear and clothing, vehicles, crates, farm equipment and other materials is not carried out properly, infection will persist in the avian population and the concurrent damage to the poultry industry and the public health threat will not be halted. For this reason, cleaning and disinfecting must be considered an essential part of AI control programmes.

The possibility of reoccurrence of the AI outbreaks in Pakistan is still there because vaccination against the AIV is not rigorously practiced. This threat of the avian influenza has necessitated the pervasive use of disinfectants effective against wide range of viruses, bacteria and fungal spores. There is a wide variety of disinfectants available in market which are claimed to be effective against pathogens. The information about the efficacy of physical and the chemical (disinfectants) agents is scanty. This study, therefore, was designed to evaluate the efficacy of various physical (temperature, Ultraviolet light and pH), and chemical (commercially available disinfectants) agents against local strain of AIV H5N1. The results of this study would be helpful in implementing effective bio-security measures at the farm and hatcheries level.

## Methods

### Source of Virus

Avian influenza virus was isolated from infected poultry flocks during recent AI outbreaks in and around Rawalpindi/Islamabad area of Pakistan during 2006 at Disease Section of Poultry Research Institute (PRI), Rawalpindi, Pakistan. Subtyping as H5 was performed by haemagglutination (HA) and haemagglutination inhibition (HI) tests using specific antiserum against H5N1 (Weybridge, UK) as described by Olsen *et al. *[10]. Molecular characterization as H5N1 was carried out at National Reference Laboratory for Poultry Diseases, Animal Sciences Institute, National Agricultural Research Centre, Islamabad, Pakistan. The virus cultivated in 9–11 day-old embryonated chicken eggs was subjected to virus titration by the method of Reed and Muench [11]. The amnio-allantoic fluid (AAF) having virus titer of 10^8.3 ^ELD_50_/ml was stored in aliquot at -70°C till further use.

### Treatment of AIV H5N1 with physico-chemical agents

The preserved virus was cultivated in 9 to11-day-old embryonated chicken eggs. Harvested amnio allantoic (AAF) fluid was titrated on the basis of haemagglutination (HA) potential. Peptone water was prepared, autoclaved and incubated at 37°C for 24 h to check sterility. AAF was diluted in peptone water to have 4 HA unit titer. It was divided into aliquots in sterilized glass vials with 4 ml each. Each vial with H5N1 virus suspension was exposed to 4, 28 and 56°C, ultraviolet light, and different pH values (1, 3, 5, 7, 9, 11 and 13) for different time intervals. The disinfectants used for inactivation of the H5N1 virus included Formalin (Formaldehyde; Merck), Phenol crystals (Merck), Iodine crystals (Merck), CID 20 (CID LINES^®^, Belgium), Virkon^®^-S (Antec™ International, UK), Zeptin 10% (Nawan laboratories, Pakistan), KEPCIDE 300 (KEPRO B.V., Holland), and KEPCIDE 400 (KEPRO B.V., Holland), which were mixed with peptone water to attain the required concentration. Each disinfectant product was put in contact with virus suspension at initial concentration of 4 HA units in a ratio of 1:2 at 28°C for 15, 30, 45 and 60 minutes. Effect of soap, detergent and alkali on infectivity of H5N1 virus was also determined using Lifebuoy (Uniliver Pakistan Ltd.), Surf Excel (Uniliver Pakistan Ltd.) and Caustic Soda (Sodium hydroxide, Merck) respectively with the aforementioned protocol.

### Inoculation in chicken embryos

Each of the virus suspension exposed to physical factors or disinfectants was filtered through 0.22 μm filter (Milliplex™, Millipore corp., Bedford USA) and four chicken embryonated eggs (9 to11 day-old) were inoculated with 0.2 ml of each of the filtrate through allantoic route. Embryonated eggs were also inoculated with untreated AIV H5N1 suspension (4HA titer) and normal saline as positive and negative control respectively. Eggs were incubated at 37°C and were candled after every 24 h for consecutive 72 h. The allantoic fluid was harvested from each of the egg and tested by HA and HI as described by Olsen *et al. *[10]. The inactivation of the virus by physical and chemical treatment was indicated by the survival of the embryo and lack of HA activity of the AAF.

## Results

Avian influenza virus H5N1 retained its infectivity at 4°C for more than 100 days although HA activity was decreased. Virus lost its infectivity after 24 h when kept at room temperature (28°C). Virus tolerated 15 min exposure to 56°C however it was inactivated at 56°C after 30 min of exposure. Ultraviolet light had no deleterious effect on the virus replicating ability even after 60 minutes of exposure (Table 1).

**Table 1:** Effect of temperature and ultraviolet light on the survival of avian influenza virus H5N1 subtype

	**Exposure time (minutes)**			
**Physical factors (n = 2)**	**15**	**30**	**45**	**60**
Temperature (56°C)	++++	----	----	----
Ultraviolet light	++++	++++	+++-	++--

++++ = AAF from all four inoculated chicken embryos showed haemagglutination (HA) and haemagglutination inhibition (HI) tests positive;

------- = AAF from all four inoculated chicken embryos showed undetectable haemagglutination (HA) activity.

It was observed that H5N1 subtype lost its viability when exposed to pH 1, 3, 11 and 13 after 6 h while it remained viable at pH 7 for all contact times (6, 12, 18 and 24 h). It retained its virulence at pH 5 for 18 h but got inactivated after 24 h. Virus retained its infectivity at pH 9 for more than 24 h (Table 2).

**Table 2:** Effect of pH on the survival of avian influenza virus H5N1 subtype

	**Exposure time (h)**			
**pH Values** **(n = 6)**	**6**	**12**	**18**	**24**
1	----	----	----	----
3	----	----	----	----
5	++++	++++	+++-	----
7	++++	++++	++++	++++
9	++++	++++	++++	++++
11	+---	----	----	----
13	----	----	----	----

++++ = AAF from all four inoculated chicken embryos showed haemagglutination (HA) and haemagglutination inhibition (HI) tests positive;

------ = AAF from all four inoculated chicken embryos showed undetectable haemagglutination (HA) activity.

The results revealed that AIV H5N1 can be inactivated by disinfectants at the recommended concentrations (Table 3). H5N1 was inactivated with formalin (0.2, 0.4 and 0.6% after 15 minutes), Iodine crystals (0.4 and 0.6% after 15 minutes), Phenol crystals (0.4 and 0.6% after 15 minutes), CID 20 (0.5% after 60 minutes and 1.0% after 15 minutes), Virkon^®^-S (0.2% after 45 minutes, 0.5 and 1.0% after 15 minutes), Zeptin 10% (0.5% after 45 minutes, 1% after 30 minutes and 2% after 15 minutes), KEPCIDE 300 (0.5% after 30 minutes and 1% after 15 minutes) and KEPCIDE 400 (0.5 and 1.0% after 15 minutes) at 28°C. Lifebuoy, Surf Excel and Caustic soda inactivated the virus at 0.1, 0.2 and 0.3% concentration after 5 minutes contact time while a concentration of 0.05% was not enough to kill virus (Table 4).

**Table 3:** Effect of chemical factors on the survival of avian influenza virus H5N1 subtype

		**Exposure time (minutes)**			
**Disinfectant**	**Concentration (%)**	**15**	**30**	**45**	**60**
Formalin	0.2	----	----	----	----
	0.4	----	----	----	----
	0.6	----	----	----	----
Iodine crystals	0.2	++++	++++	++++	++++
	0.4	----	----	----	----
	0.6	----	----	----	----
Phenol crystals	0.2	++++	++++	++++	++++
	0.4	----	----	----	----
	0.6	----	----	----	----
CID 20	0.2	++++	++++	++++	++++
	0.5	++++	++++	++++	----
	1.0	----	----	----	----
Virkon^®^-S	0.2	++++	++++	----	----
	0.5	----	----	----	----
	1.0	----	----	----	----
Zeptin 10%	0.5	++++	++++	----	----
	1.0	++++	----	----	----
	2.0	----	----	----	----
KEPCIDE 300	0.2	++++	++++	++++	++++
	0.5	+++	----	----	----
	1.0	----	----	----	----
KEPCIDE 400	0.2	++++	+++	++++	++++
	0.5	----	----	----	----
	1.0	----	----	----	----

++++ = AAF from all four inoculated chicken embryos showed haemagglutination (HA) and haemagglutination inhibition (HI) tests positive;

------ = AAF from all four inoculated chicken embryos showed undetectable haemagglutination (HA) activity.

**Table 4:** Effect of soap, detergent and alkali on the survival of avian influenza virus H5N1 subtype

		**Exposure Time (minutes)**			
**Disinfectant**	**Concentration (%)**	**5**	**15**	**30**	**45**
Surf Excel^®^	0.05	++++	++++	++++	++++
	0.1	----	----	----	----
	0.2	----	----	----	----
	0.3	----	----	----	----
Life buoy^®^	0.05	++++	++++	++++	++++
	0.1	----	----	----	----
	0.2	----	----	----	----
	0.3	----	----	----	----
Caustic soda	0.05	++++	++++	++++	++++
	0.1	----	----	----	----
	0.2	----	----	----	----
	0.3	----	----	----	----

++++ = AAF from all four inoculated chicken embryos showed haem-agglutination (HA) and haem-agglutination inhibition (HI) tests positive;

------ = AAF from all four inoculated chicken embryos showed undetectable haemagglutination (HA) activity.

## Discussion

Persistence of AIV H5N1 is inversely proportional to temperature and it is evident from the data presented in this study. Virus could survive more than 100 days at 4°C but was inactivated after 24 h at 28°C and after 30 min at 56°C. Results from the two highly pathogenic avian influenza (HPAI) H5N1 viruses from Asia indicated that these viruses did not persist as long as the wild-type AIVs. The persistence of HPAI H5N1 viruses from Asia provided some insight into the potential for these viruses to be transmitted and maintained in the environments of wild bird populations [12]. There is variation in thermo stability of H5N1 viruses. Therefore quite contentious results from various parts of the world are reported. Songserm *et al. *[13] studied the stability of H5N1 HPAI virus isolated in Thailand determining the survival of the infectious virus (initial titer of 10^6.3 ^ELD50/ml) mixed with chicken faeces under different environmental conditions. It was concluded that virus completely inactivated within 30 min after direct sunlight exposure at an environmental temperature of 32 to 35°C but infectivity was still retained after 4 days in shade at 25 to 32°C. They further reported inactivation of same virus after exposure for 3 min at 70°C. Beard *et al. *[14] incubated wet faeces from naturally infected hens during the HPAI (H5N2) 1983–1985 Pennsylvania outbreak at 4 and 25°C. At 4°C infectivity could still be detected after 35 days but after incubation at 25°C only after 2 days.

Effect of heat treatment on HPAI virus (A/chicken/Korea/ES/2003, H5N1 subtype) in chicken meat was investigated by Swayne [15]. The initial titers of infected thigh and breast meat with the H5N1 strain were 10^6.8 ^and 10^5.6 ^ELD50/g, respectively. After exposure at 30, 40, 50 and 60°C (1 min), the titer in both types of meat sample remained unchanged. Complete inactivation was only reached after exposure at 70°C (1 sec) and at 70°C for 5 sec in the breast and thigh meat, respectively. The exact mechanism of heat mediated virus inactivation is not known. It is however expected that physical factors like temperature are responsible for decreasing the polymerase activity of the virus which ultimately affects its replication activity [[16] and [17]].

Previously ultraviolet radiation (UV) light has not been proven to inactivate AIVs in a timely manner, as data have shown that 45-min exposure to a UV source was not sufficient for absolute inactivation of HPAI strain A/chicken/Pakistan/94 (H7N3) at an initial concentration of 4 HA units in peptone water at pH 7 [18]. Similar results were obtained by Chumpolbanchorn *et al. *[19] who studied the effect of UV light on infectivity of avian influenza virus (H5N1, Thai field strain) in chicken fecal manure. AIV at initial concentration of 2.38 × 10^5.25 ^ELD50 was exposed to ultraviolet light at 4–5 microw/cm^2 ^at room temperature. UV light could not destroy the infectivity of the virus completely even after exposure for 4 h. Distance from the source of light and shallowness of the exposed suspension are also contributing factors in UV mediated viral destruction. Therefore, only microbes on the surface of material and in the air are killed by UV light [20].

Orthomyxoviridae are considered to be sensitive to acid pH values, although their retention of infectivity is dependent on degree of acidity and virus strain [21]. The mechanism by which AIVs infectivity is lost has been well studied. It has been reported that incubation of Influenza virus at pH 5 favors virus fusion with host cell membrane [22]. A low pH affects haemagglutinin protein which allows fusion with host cell membrane. The conformational change is reversible between pH 6.4 and 6 but irreversible below pH 5 [23]. Results of present study are partially in agreement with Sato *et al*. [23] as H5N1 virus lost its infectivity at pH below 5 (1 & 3) but remained viable even after 18 h at pH 5. Conducting similar studies, Mittal *et al. *[24] calculated the pKa (the pH value at which 50% of HA is activated) and the pKi (the pH value at which 50% of HA is inactivated) and have shown that the pKa was 5.6–5.7 and the pKi was 4.8–4.9 for H1N1 and H2N2 respectively. Hence it can be assumed that haemagglutinin of H5N1virus under investigation could not attach itself to host (Embryo) cell membrane at pH below 5 and ultimately did not replicate to survive. Similarly, Lue *et al. *[25] observed that LPAI subtypes of H7N2 lost 100% infectivity at pH 2 after 5 min, but exposure to pH 5, 7, 10 and 12 for 15 min had no effect on the infectivity of the isolates. The threshold pH, at which the infectivity is lost, depends on the haemagglutinin (HA) subtype of the virus strain. Strains with noncleaved HA are much more stable when compared to strains with cleaved HA. These observations might explain why duck influenza viruses spread well by lake water, while highly pathogenic strains with cleaved HA do not [26].

Commercially available disinfectant products are usually composed of aldehydes, oxidizing agents, phenol compounds, quaternary ammonium compounds (QACs) and alcohols. Each commercial preparation is the result of careful formulation and any modification can reduce the efficacy. Disinfectants evaluated in this study including CID-20, Virkon^®^-S, Zeptin 10%, KEPCIDE 300 and KEPCIDE 400 were effective in completely destroying H5N1 virus at recommended dilutions of 1.0, 1.0, 2.0, 1.0 and 1.0% respectively after 15 min at 28°C. Virkon^®^-S and KEPCIDE 400 were equally good in inactivating the virus at half (0.5% after 15 min) of the recommended dilution. Disinfectant induced inactivation of AIV has been reported by various researchers all over the world. Muhammad *et al*. [18] reported the efficacy of Virkon-S against H7N3 subtype and found that 0.5% dilution was able to inactivate AIV fully after 90 min while 1% and 2% concentration achieved virucidal activity in just 30 min. They further described that phenol crystal at 0.2% and 0.4% dilution required 18 and 12 h respectively to kill the same virus which is contrary to present study findings where phenol crystal at 0.4% took only 15 min to kill H5N1 at 28°C. Ito *et al*. [27] has reported the effect of six povidone iodine products at 2, 0.5, 0.25, and 0.23% concentrations on HPAI A/crow/Kyoto/T2/04 (H5N1). The results showed virucidal activity at all concentrations reducing the virus infectious titers to levels below the detection limits of virus isolation only after 10 s at 25°C. It is not in agreement with our finding where Iodine crystals at 0.2% dilution were not able to inactivate H5N1 virus even after 60 min but 0.4 and 0.6% inactivated after 15 min at 28°C. Conducting similar studies, King [28] drew a conclusion that formalin at low concentration such as 0.04% and 0.1% was able to inactivate HPAI and LPAI viruses (H5N2, H5N9 and H9N2) after 16 h at 37°C. Similar results were obtained by Muhammad *et al. *[18] who reported that 0.06% and 0.12% concentration of formalin was not sufficient to inactivate AIV H7N3 after 6 h however at a concentration of 0.24% no virus was detected by virus isolation. A time span of 12 h was necessary to inactivate AIV at all tested concentrations. These time kill studies have revealed that an inverse relationship exists between formalin concentration and required time to kill AIV of any subtype as it is evident from present study that a high concentration (0.2, 0.4 and 0.6%) of formalin killed H5N1 only after 15 min at 28°C. However, the extent of virus infectivity to be destroyed by disinfectants also depends upon the strain of the virus, exposure time, quantity of the virus and nature of the medium used.

Specific studies on the efficacy of soap, detergents and alkalis are not available in the literature. This is perhaps the first report on the efficacy of soaps, detergents and alkalis against AIVs as disinfectant. Soap and detergents are surfactants and have effect on lipid envelop of viruses which make them good disinfectant [29]. In present study, soap (Life buoy) and detergent (Surf Excel) at 0.05% concentration could not kill H5N1 virus after 45 min contact time but inactivated after 5 min at 0.1, 0.2 and 0.3% concentrations. Presence of hydroxide ion (OH^-^) in alkalis make the basis for their disinfectant activity as protein denaturation occurs. Their efficacy in denaturing protein is related to environmental temperature and is low at low temperature but increases proportionally by increasing both temperature and concentration [30]. In present study, 0.05% concentration of Caustic soda at 28°C was not sufficient in killing H5N1 virus but increasing concentrations (0.1, 0.2 and 0.3%) inactivated the virus within 5 min contact time at the same temperature (28°C).

This study describes the effects of physical and chemical agents on infectivity of AIV H5N1. It is therefore inferred that H5N1 virus can be inactivated in the poultry farms/hatcheries using high temperature (e.g. 56°C or above), low (1 and 3) or high (11 and 13) pH of the material to be disinfected. However, it may not be practically feasible for the farmers. Use of disinfectants seems more appropriate and practicable. Consequently there is no need to depopulate the poultry sheds after AIV outbreak for long period of time before arrival of new stock if disinfectants are used appropriately.

## Competing interests

The authors declare that they have no competing interests.

## Authors' contributions

MAS and MA participated in the design of the study and performed the investigation, analysis and interpretation of data. MAS and SHas conceived of the study, and participated in its design and coordination. MAS, MA and SHam drafted the manuscript while MA also working as corresponding author. All authors read and approved the final manuscript.
